# The Psoriasis Disability Index in Romanian Psoriasis Patients during COVID-19 Pandemic: Contribution of Clinical and Psychological Variables

**DOI:** 10.3390/jcm12186000

**Published:** 2023-09-16

**Authors:** Carina Mihu, Codruța Alina Popescu, Maria Adriana Neag, Ioana Corina Bocşan, Carmen Stanca Melincovici, Adrian Lucian Baican, Bogdana Adriana Năsui, Anca-Dana Buzoianu

**Affiliations:** 1 Department of Pharmacology, Toxicology and Clinical Pharmacology, Iuliu Haţieganu University of Medicine and Pharmacy, 400012 Cluj-Napoca, Romania; carina.mihu@umfcluj.ro (C.M.); maria.neag@umfcluj.ro (M.A.N.); bocsan.corina@umfcluj.ro (I.C.B.); abuzoianu@umfcluj.ro (A.-D.B.); 2 Department of Human Sciences, Iuliu Haţieganu University of Medicine and Pharmacy, 400012 Cluj-Napoca, Romania; 3 Department of Histology, Iuliu Haţieganu University of Medicine and Pharmacy, 400012 Cluj-Napoca, Romania; carmen.melincovici@umfcluj.ro; 4 Department of Dermatology, Iuliu Haţieganu University of Medicine and Pharmacy, 400012 Cluj-Napoca, Romania; abaican@umfcluj.ro; 5 Department of Cummunity Medicine, Iuliu Haţieganu University of Medicine and Pharmacy, 400012 Cluj-Napoca, Romania; adriana.nasui@umfcluj.ro

**Keywords:** quality of life, psychological variables, psoriasis

## Abstract

Background: Psoriasis is one of the most frequent chronic inflammatory skin diseases and has a negative impact on the interpersonal relationship and psychosocial well-being. The aims of this study were to examine the effects of intensity of pruritus on quality of life and depression, to investigate the relationship between anger, self-esteem, and depression, and to compare patients with early and late onset of psoriasis. As our study was carried out during the COVID-19 pandemic, we aimed also to investigate the safety concerns and anxiety related to COVID-19 in psoriasis patients. Methods: This cross-sectional study included 137 patients diagnosed with plaque psoriasis. The patients were classified as early-onset (age < 30 years) and late-onset psoriasis (age ≥ 30 years). Duration of disease, pruritus scores, and socio-demographic characteristics were recorded. Measures included the State-Trait Anger Expression Inventory (STAXI), Rosenberg Self-Esteem Scale, Beck Depression Inventory (BDI-II), Psoriasis Disability Index (PDI), and Fear and anxiety in relationship with COVID-19 Scale were used for determining anger, anger expression style, self-esteem, depression, anxiety, and quality of life. Results: The psoriasis patients had a lower score for self-esteem than the normative data from the Romanian general population. The average scores for state anger and trait anger are similar to the normative data from the Romanian general population, but the scores for anger-in and anger-out are higher. Patients with early onset had higher depression scores and lower quality of life. Self-esteem correlates negatively with depression, anger, severity of disability due to psoriasis, number of affected areas, and duration of disease. Lower level of self-esteem led to increased anger. Conclusions: Reduced self-esteem, increased anger levels, and depression are present in psoriasis patients. The effective treatment of psoriasis must, therefore, consist of a multidisciplinary approach, in which the personalized treatment of the skin condition is as important as the adjuvant therapies that reduce the patients’ stress level.

## 1. Introduction

Psoriasis, a commune and persistent immune-mediated skin condition [1], has an estimated prevalence of approximately 2% of the global adult population [2,3] and affects men and women equally [4]. The reported prevalence of psoriasis in Romania is 5.18% [5,6]. Although onset of psoriasis may occur at any age from early infancy to old age, it typically starts in 60% of patients before the age of 30 and in 14% before the age of 10 [4,7,8]; consequently, chronicity, the requirement for ongoing medical attention, and its impact on physical appearance can undoubtedly affect the mental health and quality of life of the patients.

In terms of the causes and worsening of psoriasis, psychological stressors play a significant role by triggering the excessive activation of the hypothalamic–pituitary–adrenal axis [9]. This, in turn, leads to an elevated release of proinflammatory cytokines, exacerbating the condition [10]. Conversely, the inflammatory response in the skin can potentially trigger symptoms of anxiety and depression [11], primarily due to changes in physical appearance and the perception of being stigmatized [12]. Stigmatization tends to be more prevalent among individuals with psoriasis compared to those with other dermatological conditions [13]. This phenomenon often results in diminished levels of self-esteem among psoriasis patients [14,15].

Quality of life encompasses various dimensions, including physical, social, and emotional well-being. The negative impact of psoriasis on a patient’s quality of life is well documented in the literature, highlighting the understanding that the psychological and social implications of psoriasis are of equal significance to its physical effects, contributing to the overall morbidity of the disease [16,17,18,19,20]. Psoriasis Disability Index (PDI) is a disease-specific instrument, with a high face validity containing questions perceived by patient as relevant to them [21].

Patients with psoriasis are 1.5 times more prone to experiencing symptoms of depression [22]. The relationship between a patient’s mental health status and skin diseases is bidirectional; therefore, it is essential to assess how psychological factors impact a patient’s quality of life (QoL) [23]. The prevalence of comorbid clinical depression (12–19%) and anxiety (7–16%) among patients with psoriasis is significantly higher than among healthy controls [24], with the severity of psoriasis or the visibility of plaques [25,26,27] and pruritus [28] being linked to the depression. Self-esteem is closely associated with physical appearance and dissatisfaction with body image may lead to anger in these patients [29,30]. Anger serves as an emotional reaction to unmet wishes, undesirable outcomes, and expectations that have not been satisfied. This response is integral for the ongoing process of life. The most beneficial approach to manage anger involves its expression without resorting to aggression. When anger is unable to be expressed in a constructive manner, it can eventually turn inward and contribute to the emergence of psychosomatic disorders or mental health conditions such as depression. The suppression of underlying animosity and anger has been recognized as a significant contributor to the development of somatization [31,32]. Anger frequently accompanies psycho dermatologic diseases such as psoriasis [29,30]. The COVID-19 pandemic has caused a global public health emergency resulting in unprecedented individual and societal fear and anxiety [33]. The anxiety associated with the actual COVID-19 infection, as well as with the isolation and social distancing that people have to experience due to the pandemic, can be a potential cause for exacerbation of several chronic inflammatory skin conditions, especially those which are stress-induced, such as psoriasis [34]. Compared to patients with other diagnoses, patients with psoriasis experienced higher anxiety and more frequent concerns about the safety of their treatment [35]. Psoriasis patients can also experience cutaneous side effects after COVID-19 vaccination [36], and fear of side effects of the vaccine is one of the components of vaccine hesitancy, even in the general population [37].

The aims of this study were to:

1. Examine the effects of intensity of pruritus on disease burden and mental health outcome in patients with psoriasis.
2. Investigate the relationship of anger state and trait, anger control, and anger expression style with level of self-esteem in patients with psoriasis and to find out whether the duration and severity of the disease have an effect on the anger and anger expression style and self-esteem.
3. Compare the level of self-esteem and depression in patients with early and late onset of psoriasis.
4. Assess the safety concerns and anxiety in psoriasis patients during the COVID-19 pandemic.

## 2. Materials and Methods

### 2.1. Participants

This cross-sectional study was conducted over a 12-month period from 1 July 2021 to 1 July 2022.

All study participants were recruited and enrolled in our survey consecutively in the order they visited the dermatology outpatient clinic of the Department of Dermatology and Venereology of the County Emergency Hospital the Cluj Napoca, Romania.

Inclusion criteria were plaque psoriasis diagnosed by a dermatologist, age of 18–65 years (men and women), good level of Romanian language, at least 4 years of education and ability to read, and no serious mental or cognitive disturbances. 

A total of 150 patients were approached during the routine visits to the hospital. Of these, 137 agreed to participate (91.33%) and gave their written informed consent. The study was approved by the ethics committee of the University of Medicine and Pharmacy ‘’Iuliu Hațieganu’’ Cluj Napoca (approval number: 240/30 June 2021).

### 2.2. Measurements

A standardized form was used to collect information on demographic data, such as sex, age, marital status, education level, occupation, and place of residence, and clinical information (duration of the disease, family history of psoriasis, and smoking and drinking behaviors).

#### 2.2.1. The Psoriasis Disability Index (PDI)

The quality of life of psoriasis patients was measured with the Romanian version [38] of the Psoriasis Disability Index (PDI) [39].

This questionnaire is a 15-item self-report scale and can be handed over to the patient who is asked to fill it in without the need for a detailed explanation. It is usually completed in 3 or 4 min. The PDI total score is calculated by summing the score of each of the 15 questions, resulting in a maximum of 45 and a minimum of 0. The higher the score, the more the QOL is impaired. The Psoriasis Disability Index also has five subscales: Daily activities, Work or school or alternative questions, Personal relationships, Leisure, and Treatment.

#### 2.2.2. Severity of Pruritus

Itching was evaluated using a visual analog scale (VAS). The patients were asked to rate the severity of the pruritus that they were experiencing in the last week before they were completing the study questionnaire. The patients with psoriasis rated the “Itching due to psoriasis” on a 5-point scale from “1” denoting “not at all” and a rating of “5” denoting “very severe”.

#### 2.2.3. State Trait Anger Expression Inventory (STAXI)

Anger was assessed using the Romanian version of the State Trait Anger Expression Inventory (STAXI) [40].

The State-Trait Anger Expression Inventory (STAXI) has five main scales as well as two additional scales. The state anger scale (S-A) records the intensity of the subjective state of anger at a point in time or in a defined situation. The trait anger scale records the interindividual differences in disposition to react with an increase in state anger when anger has been provoked in a certain situation. The scale to record anger targeted at oneself (anger-in) measures the frequency with which angry feelings are repressed or not expressed openly. The scale to record anger expressed openly (anger-out) measures the frequency with which an individual expresses anger towards people or objects in his environment. The anger control scale is an indicator for the frequency with which anger is controlled or repressed.

STAXI is a well-established self-rating scale with high stability and validity, often used in clinical research [41].

#### 2.2.4. Beck Depression Inventory (BDI-II)

Depression was assessed using Romanian version of Beck Depression Inventory (BDI-II) [42].

The Beck Depression Inventory (BDI-II) is one of the most widely used self-report measures of depression in both research and clinical practice, with high validity and good psychometric properties [43]. The questionnaire consists of 21 items, and answers are rated on a four-point scale (0 = low, 3 = high). The total score ranges from 0 to 63. For persons who have been clinically investigated for depression, scores from 0 to 9 indicate that a person is not depressed, 10 to 18 indicate mild–moderate depression, 19 to 29 indicate moderate–severe depression, and 30 to 63 indicate severe depression [43,44].

#### 2.2.5. The Rosenberg Self-Esteem Scale

Self-esteem was measured using the Romanian version of the Rosenberg Self-Esteem Scale [45]. The Rosenberg Self-Esteem Scale [46] is a widely used self-report instrument for evaluating individual self-esteem and consists of a 10-item scale that measures global self-worth by measuring both positive and negative feelings about the self. All items are answered using a 4-point Likert scale format ranging from strongly agree to strongly disagree.

#### 2.2.6. Fear and Anxiety in Relationship to COVID-19

We used two questionnaires to assess the COVID-19-related anxiety and fear that were validated in a previous study [47]. The first questionnaire has 12 items and was adapted after Ho et al. [48] and assessed the participants’ opinion via a 4-point Likert scale: 0—definitely false, 3—definitely true. The second one was adapted after Ahorsu et al. [49], has 7 items, and the answers are given via a 5-point Likert scale: 1—strongly disagree, 5—strongly agree.

### 2.3. Statistical Analysis

The statistical analysis was performed using SPSS, version 24 for Windows. Categorical variables were reported with frequency and percentage, and continuous variables were illustrated by the mean and standard deviation. The normality of the distributions of the data was first assessed by Shapiro–Wilk’s tests. Because the data were not normally distributed, between-group comparisons of continuous variables were performed using Mann–Whitney U-test. Correlation analysis was performed using Pearson’s correlation coefficient. The results were considered statistically significant if the *p*-value was less than 0.05.

## 3. Results

A total of 137 patients with psoriasis were included in the study and consisted of 79 men (57.7%) and 58 women (42.3%) with a mean age of 50.27 ± 13.77 years (range: 20–65 years). The mean duration of the disease was 18.89 ± 15.32 years.

Table 1 shows sociodemographic characteristics of the participants; data are expressed as mean ± standard deviation and as number (percentage).

About half of the patients (57.2%) resided in the city. 

Further, 25.5% of the patients had secondary education, 20.5% had graduated from high school, and 32.8% had a university degree. Most of the patients (62.9%) were married or in a relationship (10.2%) and 87.1% had children.

Table 2 shows clinical characteristics of the participants; data are expressed as mean ± standard deviation and as number (percentage).

A total of 23.6% of the patients were smokers or previous smokers (52.6%) and 57.7% of them drank alcohol.

Concomitant disease was present in some of the patients and was most commonly hypertension (46.7%) and diabetes (16.1%).

In all individuals, psoriasis had been diagnosed before entry into the study. The mean (±SD) duration of psoriasis (the time elapsed between the first diagnosis of psoriasis and entry into the study) was 18.89 (±15.32) years.

A familial history of psoriasis was elicited in 16.1% of cases.

The average of total PDI was 8.65 (±5.94), whereas the average of PDI components were 4.39 (±2.83) for daily activities, 1 (±1.08) for work or school, 1.1 (±1.48) for personal relationships, 1.99 (±2.1) for leisure, and 0.2 (±0.51) for treatment.

Table 3 shows the average scores for the psychological variables.

The correlations between clinical and psychological variables of psoriasis patients are shown in Table 4.

Self-esteem correlates negatively with depression, anger, severity of disability due to psoriasis, number of affected areas, and duration of disease.

Depression is positively correlated with disease duration, severity of disability, and COVID-19-related anxiety.

Number of affected areas correlates positively with anger and disease duration.

Anger is correlated with number of affected areas, duration of illness, and COVID-19-related anxiety.

Table 5 presents the comparisons of quality if life and psychological variables based on gender.

Women scored higher on depression. Quality of life was lower in women. Compared to women, men had higher scores for anger (as a condition and trait). Women had more control over their anger. There were no differences between men and women in anxiety and fear related to COVID-19.

Table 6 shows the impact of the number of areas affected by psoriasis on quality of life, depression, anger, self-esteem and anxiety.

Patients who have had more areas affected by psoriasis have lower quality of life and self-esteem. No differences were found between anger as a condition and trait according to the number of affected areas. Patients with more areas affected by psoriasis had lower anger expression scale scores (anger-in and anger-out) and higher anger control scale scores (anger control internal and anger control internal external). COVID-19 Fear Score was higher in patients who had more areas affected by psoriasis.

Table 7 shows the effect of severity of itching on quality of life and psychological variables.

Patients with higher severity of itching had higher depression scores. Both anger as a condition and as a trait are higher in patients with more severe itching. Internal expression of anger is higher and external expression lower in these patients. Patients with higher severity of itching had higher BMI.

Table 8 shows the differences on clinical and psychological variables in psoriasis patients with early/late onset.

Patients with early onset had higher depression scores, lower quality of life, and higher COVID-19 anxiety. Anger-in, anger-out, anger control external and anger expression index are higher in patients with early onset. COVID-19 anxiety is higher in patients with early onset.

Table 9 presents the impact of depression on quality of life, anxiety and anger.

Patients with depression (BDI scores >13) had lower quality of life and self-esteem. Anger as a trait and internal anger control were lower in these patients.

We used item 16 of the BDI questionnaire to classify patients according to the presence/absence of sleep disturbances; the results are presented in Table 10.

Patients with sleep problems had lower self-esteem than patients without sleeping problems and scored higher on depression.

## 4. Discussion

Psoriasis patients face in their daily life stigmatization due to a rejection reaction from the general population, which, having no medical knowledge and being afraid of contamination, avoid contact with these patients. This aspect has a strong impact on patients with psoriasis, significantly reducing social integration. The impact is even stronger in the case of young adults who want a more active social life. Deficient social integration causes a decrease in self-esteem and the appearance of depression. Depression is accentuated by the reduction in couple relationships in these patients, who tend to isolate and avoid couple relationships.

The aims of this study were to examine the effects of intensity of pruritus on quality of life and depression, to investigate the relationship between anger, self-esteem, and depression, and to compare patients with early and late onset of psoriasis. As our study was carried out during the COVID-19 pandemic, we aimed also to investigate the safety concerns and anxiety related to COVID-19 in psoriasis patients.

In our sample, the average duration of the disease was 18.89 ± 15.32 years; the high standard deviation is explained by the characteristics of our sample of psoriasis patients living in community in our region, in which 62.77% of the patients had an onset of psoriasis before the age of 30.

The psoriasis patients had a mean score for self-esteem of 27.39 (±4.62), which is a lower score; normative data from the Romanian general population showed that 69% of the population scored higher then 30 on self-esteem [45].

The average scores for state anger and trait anger are similar with normative data from the Romanian general population, but the scores for anger-in and anger-out are higher [40].

Self-esteem correlates negatively with depression, anger, severity of disability due to psoriasis, number of affected areas, and duration of disease. Lower level of self-esteem led to increased anger. The results are similar to those of Aydin [30], who found state anger and trait anger higher in patients with psoriasis compared to the healthy group, and to those of Conrad [50], who also found that trait anger, state anger, anger-in, and anger-out levels are higher in patients with psoriasis than in control groups.

In total, 24.1% of the patients had depression. Depression is positively correlated with disease duration, severity of disability, and COVID-19-related anxiety. Patients with depression had lower quality of life and self-esteem. Similar findings have also been demonstrated by other authors, who found that psoriasis patients were more likely to be more depressed than the general population controls and that scores for depression were correlated with the severity of the psoriasis symptoms [22,25,26]. Pruritus is one of the most bothersome subjective symptoms of psoriasis; in our study, patients with higher severity of itching had higher depression scores, higher BMI, and are prone to expressing their anger internally. A lot of studies had shown the impact of pruritus on quality of life in psoriasis patients [51,52].

Patients with early onset had higher depression scores and lower quality of life, results similar with another study [53]. A possible explanation for the differences between the patients with early/late-onset psoriasis regarding the level of depression is the relationship between body image, social anxiety, and depression [54]. Psoriatic lesions may trigger negative body image emotions, such as shame, and this could lead to low self-esteem and depression, especially in young adults who are more focused on their image [55], leaving them psychologically vulnerable and prone to experiencing depression.

Nonadherence to treatment and lacking access to care is a pivotal risk factor for the aggravation of psoriasis, which can increase patient distress, disability, and diminished quality of life. In our study, 94.9% of the patients had no problems being seen by dermatologists during the pandemic. A total of 37.2% of the patients had COVID-19 infection. The percentage of patients vaccinated was 58.4%, similar with the vaccination percentage in Cluj County (58.48%) and higher than the general population in Romania (46.3%).

Patients who had an earlier onset of psoriasis had more anxiety related to COVID-19 and were also older. Mean COVID-19-related anxiety scores are similar to those in another study [47]. Another study conducted in Romania during the pandemic (but on a small group of patients) shows that the patients prone to developing anxiety during the pandemic were women, patients over the age of 65, those in urban areas, alcohol users, non-smokers, and those with moderate forms of psoriasis [56].

## 5. Conclusions

The results of our study, carried out during the COVID-19 pandemic, showed that lower-level quality of life, reduced self-esteem, increased anger levels, and depression are present in psoriasis patients. Patients with early-onset psoriasis had higher depression scores and lower quality of life. In all the patients, self-esteem was negatively correlated with depression, anger, and psoriasis disability levels.

The social inclusion of patients with psoriasis is essential for increasing their quality of life. Medical education campaigns addressed to the general population would be helpful to integrate patients with psoriasis into society. Moreover, this would lead to the reduction in stress in these patients, knowing that stress is one of the important triggering factors in the occurrence or exacerbation of psoriasis.

The effective treatment of psoriasis must, therefore, consist of a multidisciplinary approach, in which the personalized treatment of the skin condition and possible cardiac or joint complications are as important as the adjuvant therapies that reduce the patients’ stress level.

Such a dual approach to the physical and mental components of psoriasis can significantly improve the mental health, the evolution of the disease, and the social integration of these patients.

## Figures and Tables

**Table 1:** Sociodemographic characteristics of the participants.

Age (Mean ± SD) Years	50.27 ± 13.77
Sex	
Male	79 (57.7%)
Female	58 (42.3%)
Educational status	
Secondary	35 (25.5%)
High school	28 (20.5%)
College	29 (21.2%)
University	45 (32.8%)
Marital status	
Single	15 (10.9%)
In a relationship	14 (10.2%)
Married	86 (62.9%)
Divorced	15 (10.9%)
Widowed	7 (5.1%)
Residence	
Urban	72 (52.6%)
Rural	65 (47.4%)
Children	
No	30 (21.9%)
Yes	107 (78.1%)

**Table 2:** Clinical characteristics of the study population.

Psoriasis Localization (Yes, N, %)	
Face	35 (25.5%)
Scalp	101 (73.7%)
Upper limbs	122 (89.1%)
Lower Limbs	122 (89.1%)
Trunk	113 (82.5%)
Number of areas affected by psoriasis (N, %)	
1 or 2 area	23 (16.8%)
More than 2 area	114 (83.2%)
duration of the disease (mean ± SD) years	18.89 (±15.32)
Age at onset (N, %)	
Before 30 years	86 (62.77%)
After 30 years	51 (37.23%)
BMI (mean ± SD)	28.34 (±5.28)
BMI categories (N, %)	
Underweight (<18.5)	7 (5.1%)
Normal weight (≥18.5 <25)	22 (16.1%)
Overweight (≥25 <30)	65 (47.4%)
Obese (≥30)	43 (31.4%)
Comorbidities (Yes, N, %)	
Hypertension	64 (46.7%)
Diabetes	22 (16.1%)
Health risk behaviors (Yes, N, %)	
History of smoking	72 (52.6%)
Present smoking	36 (26.3%)
Drinking Alcohol	79 (57.7%)
Cigarettes/day (mean ± SD)	3.21 ± 6.33
PDI (mean ± SD)	
Daily activities	4.39 (±2.83)
Work or school	1 (±1.08)
Personal relationships	1.1 (±1.48)
Leisure	1.99 (±2.1)
Treatment	0.2 (±0.51)
PDI total	8.65 (±5.94)
Severity of itching (N, %)	
No itching	15 (10.9%)
Mild itching	2 (1.5%)
Moderate Itching	16 (11.7%)
Serious Itching	44 (32.1%)
Severe Itching	60 (43.8%)
Lifetime hospitalization for psoriasis (Yes, N, %)	84 (61.3%)
Difficulty accessing dermatological consultations during COVID-19 pandemic—Yes, N (%)	7 (5.1%)
Family history of psoriasis—Yes, N (%)	22 (16.1%)

**Table 3:** Scores for depression, anger, and self-esteem (mean ± SD).

BDI	6.1 (±5.3)
STAXI	
State anger	15.6 (±0.9)
Trait anger	17.2 (±3.7)
Anger reaction	8.1 (±2.2)
Anger verbal	5 (±0.91)
Anger physical	5 (±0.43)
Anger-in	17.7 (±3.7)
Anger-out	23.4 (±5.7)
Anger control external	23.4 (±5.7)
Anger control internal	20 (±4.3)
Anger expression index	38.18 (14.2)
Self-esteem	27.39 (±4.62)
COVID-19 Fear	7.05 (±5.09)
COVID-19 Anxiety	16.09 (±6.22)

**Table 4:** Correlation between clinical and psychological variables.

	BDI	PDI	Self-Esteem	Number of Areas Affected by Psoriasis	COVID-19 Anxiety	COVID-19 Fear	Duration of the Disease
BDI	1	0.306 **	−0.611 **	0.164	0.183 *	−0.083	0.286 **
PDI	0.306 **	1	−0.174 *	0.081	−0.096	0.008	−0.344 **
Self esteem	−0.611 **	−0.174 *	1	−0.422 **	−0.209 *	0.032	−0.312 **
Number of areas affected by psoriasis	0.164	0.081	−0.422 **	1	0.231 **	0.169 *	0.390 **
COVID-19 Anxiety	0.183 *	−0.096	−0.209 *	0.231 **	1	0.314 **	0.238 **
COVID-19 Fear	−0.083	0.008	0.032	0.169 *	0.314 **	1	0.052
State anger	−0.022	0.101	−0.241 **	0.104	0.388 **	0.285 **	−0.167
Trait anger	−0.124	0.006	−0.312 **	0.364 **	−0.149	0.396 **	0.369 **
Anger-out	−0.013	−0.001	0.165	0.369 **	0.124	−0.133	0.256 **
Anger-in	0.090	−0.003	0.079	0.528 **	0.218 *	−0.337 **	−0.060
Anger control external	−0.040	0.112	0.041	0.235 **	−0.502 **	−0.377 **	0.084
Anger control internal	−0.037	−0.002	0.084	0.232 **	−0.519 **	−0.115	0.098
Anger expression index	0.047	−0.047	0.037	0.438 **	0.465 **	0.052	0.010
duration of the disease	0.286 **	−0.344 **	−0.312 **	0.390 **	0.238 **	0.052	1

** *p* < 0.001, * *p* < 0.05.

**Table 5:** Comparison of quality of life, depression, self-esteem, anger, and pandemic anxiety by gender.

	Male (N = 79)		Female (N = 58)		*p* (Mann-Whitney U Test)
	Mean	Std. Deviation	Mean	Std. Deviation	
BDI	4.74	5.04	7.94	5.28	0.000
PDI	6.50	5.02	11.58	5.88	0.000
Self-esteem	27.54	4.65	27.18	4.61	>0.05
COVID-19 Fear	7.18	5.95	6.86	3.65	>0.05
COVID-19 Anxiety	17.22	7.54	14.5517	3.55988	>0.05
State anger	15.88	1.16	15.25	0.44	0.003
Trait anger	16.50	3.20	18.25	4.11	0.032
Anger-out	15.59	4.23	16.44	5.89	>0.05
Anger-in	17.87	4.61	17.46	2.24	>0.05
Anger control external	22.07	5.84	25.31	5.22	0.001
Anger control internal	19.25	3.82	21.08	4.76	0.005
Anger expression index	40.13	14.31	35.51	13.72	0.01

**Table 6:** Comparison of quality of life, depression, self-esteem, anger, and pandemic anxiety by number of areas affected by psoriasis.

	1 or 2 Areas Affected by Psoriasis (N = 23)		More than Areas Affected by Psoriasis (N = 114)		*p* (Mann–Whitney U Test)
	Mean	Std. Deviation	Mean	Std. Deviation	
BDI	6.26	4.03	6.07	5.61	>0.05
PDI	6.13	4.26	9.16	6.11	0.042
Self-esteem	30.00	2.52	26.86	4.78	0.001
COVID-19 Fear	4.52	4.90	7.56	5.00	0.011
COVID-19 Anxiety	16.30	4.95	16.05	6.55	>0.05
State anger	15.34	0.48	15.67	1.04	>0.05
Trait anger	17.73	1.25	17.14	4.02	>0.05
Anger-out	20.78	7.30	14.98	3.74	0.004
Anger-in	22.43	4.13	16.74	2.91	0.000
Anger control external	19.08	1.41	24.32	5.95	0.000
Anger control internal	17.26	3.20	20.58	4.32	0.001
Anger expression index	54.86	13.40	34.81	11.81	0.000

**Table 7:** Comparison of quality of life, depression, self-esteem, anger, and pandemic anxiety by severity of itching.

	Severity of Itching—Mild (N = 17)		Severity of Itching—Moderate to Severe (N = 120)		*p* (Mann–Whitney U Test)
	Mean	Std. Deviation	Mean	Std. Deviation	
BMI	21.10	5.14	29.36	4.44	0.000
BDI	5.58	2.50147	6.17	5.66	0.019
PDI	5.00	5.22015	9.17	5.87	>0.05
Self-esteem	27.17	1.28624	27.42	4.92	>0.05
COVID-19 Fear	6.00	4.63681	7.20	5.15	>0.05
COVID-19 Anxiety	13.11	3.15995	16.51	6.52	0.044
State anger	15.00	0.00000	15.70	1.01	0.001
Trait anger	15.00	1.76777	17.56	3.80	0.002
Anger-out	23.70	3.29326	14.85	4.16	0.000
Anger-in	19.41	6.58653	17.45	3.17	>0.05
Anger control external	23.70	6.47847	23.40	5.72	0.013
Anger control internal	22.35	3.23	19.70	4.37	>0.05
Anger expression index	45.05	16.43	37.20	13.65	>0.05

**Table 8:** Comparison of quality of life, depression, self-esteem, anger, and pandemic anxiety by psoriasis onset.

	Early Onset (before 30 Years)		Late Onset (after 30 Years)		*p* (Mann–Whitney U Test)
	Mean	Std. Deviation	Mean	Std. Deviation	
BDI	7.46	5.763	3.80	3.66	0.001
PDI	10.13	6.75	6.15	2.89	0.000
Self-esteem	26.76	5.56	28.45	1.94	>0.05
COVID-19 Fear	7.01	4.40	7.13	6.14	>0.05
COVID-19 Anxiety	18.41	5.94	12.17	4.78	0.000
State anger	15.58	0.86	15.68	1.15	>0.05
Trait anger	16.47	4.14	18.54	2.30	>0.05
Anger-out	17.36	4.40	13.58	5.10	0.002
Anger-in	18.22	2.62	16.82	5.10	0.000
Anger control external	23.11	4.93	24	7.04	0.000
Anger control internal	19.26	3.85	21.31	4.80	>0.05
Anger expression index	41.19	11.57	33.09	16.70	0.003

**Table 9:** Comparison of quality of life, self-esteem, anger, and pandemic anxiety in psoriasis patients with or without depression.

	No Depression (N = 104)		Depression (N = 33)		*p* (Mann–Whitney U Test)
	Mean	Std. Deviation	Mean	Std. Deviation	
PDI	7.71	4.42	11.63	8.66	0.001
Self-esteem	29.02	2.61	22.24	5.73	0.000
COVID-19 Fear	7.52	5.29	5.54	4.12	>0.05
COVID-19 Anxiety	15.44	5.52	18.15	8.05	>0.05
State anger	15.59	1.02	15.69	0.80	>0.05
Trait anger	17.55	3.27	16.27	4.74	0.035
Anger-out	16.30	5.43	14.84	3.11	>0.05
Anger-in	17.62	4.25	17.93	1.59	>0.05
Anger control external	23.52	6.28	23.18	3.95	>0.05
Anger control internal	20.33	4.58	19.06	3.26	0.047
Anger expression index	38.06	15.75	38.54	7.62	>0.05

**Table 10:** Comparison of quality of life, depression, self-esteem, anger, and pandemic anxiety by sleep quality (BDI item 16).

	No Sleep Problem (N = 98)		Sleep Problems (N = 39)		*p* (Mann–Whitney U Test)
	Mean	Std. Deviation	Mean	Std. Deviation	
BDI	3.23	2.88	13.30	2.75	0.000
PDI	7.61	4.28	11.28	8.35	>0.05
Self-esteem	28.90	2.94	23.58	5.81	0.000
COVID-19 Fear	7.39	5.43	6.17	4.04	>0.05
COVID-19 Anxiety	15.60	5.89	17.33	7.15	>0.05
State anger	15.64	1.047	15.56	0.78	>0.05
Trait anger	17.47	3.30	16.66	4.56	>0.05
Anger-out	16.11	5.51	15.56	3.41	>0.05
Anger-in	17.71	4.35	17.66	1.70	>0.05
Anger control external	23.55	6.36	23.17	4.09	>0.05
Anger control internal	20.19	4.67	19.61	3.32	>0.05
Anger expression index	38.08	16.15	38.43	7.45	>0.05

## Data Availability

The data that support the findings of this study can be requested from the corresponding author.
